# Vitamin D_3_ reduces the symptoms of ovarian hyperstimulation syndrome in mice and inhibits the release of granulosa cell angiogenic factor through pentraxin 3

**DOI:** 10.1007/s11626-024-00898-z

**Published:** 2024-04-04

**Authors:** Minping Zhang, Li Chen, Qunping Xu, Xiaohua Yang, Xiang Liu, Luanmei Liu

**Affiliations:** Department of Pharmacy Management, Nanping First Hospital Affiliated to Fujian Medical University, 317 Zhongshan Road, Nanping, Fujian 353000 People’s Republic of China

**Keywords:** Growth factor, OHSS, Ovary, Vitamin, VEGF

## Abstract

It has been reported that the effective inhibition of vascular endothelial growth factor (VEGF) can prevent the progression of ovarian hyperstimulation syndrome (OHSS). The present study aimed to investigate the mechanism underlying the effect of vitamin D_3_ (VD3) on OHSS in mouse models and granulosa cells. The effects of VD3 administration (16 and 24 IU) on ovarian permeability were determined using Evans blue. In addition, ovarian pathology, corpus luteum count, inflammatory responses, and hormone and VEGFA levels were assessed using pathological sections and ELISA. Molecular docking predicted that pentraxin 3 (PTX3) could be a potential target of VD3, and therefore, the effects of human chorionic gonadotropin (hCG) and VD3 as well as PTX3 overexpression on the production and secretion of VEGFA in granulosa cells were also investigated using western blotting and immunofluorescence. Twenty-four IU VD3 significantly reversed the increase in ovarian weight and permeability in mice with OHSS. Additionally, VD3 diminished congestion and the number of corpus luteum in the ovaries and reduced the secretion levels of inflammatory factors and those of estrogen and progesterone. Notably, VD3 downregulated VEGFA and CD31 in ovarian tissues, while the expression levels of PTX3 varied among different groups. Furthermore, VD3 restored the hCG-induced enhanced VEGFA and PTX3 expression levels in granulosa cells, whereas PTX3 overexpression abrogated the VD3-mediated inhibition of VEGFA production and secretion. The present study demonstrated that VD3 could inhibit the release of VEGFA through PTX3, thus supporting the beneficial effects of VD3 administration on ameliorating OHSS symptoms.

## Introduction

Ovarian hyperstimulation syndrome (OHSS) is a potentially life-threatening iatrogenic complication, which is commonly associated with fertility treatments (Hortu *et al.* 2021). This condition is characterized by the abrupt translocation of fluid from the vascular to the third space, triggered by the enhanced permeability of the capillaries. A previous study revealed that OHSS was associated with the presence of ovarian interstitial edema, alongside luteal follicle cysts, necrosis, and neovascularization (Cheng *et al.* 2020). The onset of OHSS is primarily associated with increased vascular permeability induced by human chorionic gonadotropin (hCG), which mediates the synthesis of pro-inflammatory mediators (Şanlı *et al.* 2023). Notably, several cytokines and angiogenic molecules, including vascular endothelial growth factor (VEGF), have been involved in the pathogenesis of OHSS (Wang *et al.* 2021). Previous studies indicated that the effective inhibition of the VEGF-related vascular permeability via targeting VEGF secretion could prevent the progression of OHSS (Hortu *et al.* 2021; Huang *et al.* 2022; Zhang *et al.* 2022).

Furthermore, other studies highlighted the potential of vitamin D in mitigating VEGF production, as evidenced by its ability to reduce VEGF levels in peritoneal mesothelial cells and several cancer cell lines (Wu *et al. *2019; Da *et al. *2020). In clinical settings, vitamin D supplementation among vitamin D–deficient women with polycystic ovary syndrome (PCOS) exhibited promising results, manifested by reduced serum VEGF levels and improved clinical symptoms of PCOS (Irani *et al.* 2017). These findings supported the potential efficacy of vitamin D as a therapeutic intervention for managing OHSS. However, the particular effect of vitamin D on VEGF expression in OHSS remains elusive, potentially due to limitations in dosing techniques and adequate therapeutic concentration (Turan *et al.* 2015). Therefore, the pathological implications and mechanisms of action of vitamin D should be further investigated. Additionally, although the regulatory role of vitamin D in various autoimmune diseases has been well-documented, its effect on OHSS remains unknown.

Furthermore, another study showed that pentraxin 3 (PTX3) was notably upregulated in OHSS (Korhonen *et al.* 2020). PTX3 silencing has been identified as a suppressor of VEGF production (Rajkovic *et al.* 2018). Molecular docking analysis highlighted the potential of vitamin D_3_ (VD3) in modulating VEGF expression through PTX3, thus emphasizing its possible involvement in the complex network underlying OHSS pathology. In the current study, mouse OHSS models and granulosa cells were utilized to reveal the involvement of PTX3 in the mechanism underlying the effect of VD3 on regulating OHSS.

## Methods and materials

### Mouse grouping and modeling

A total of 40 ICR mice (gender, female; age, 5 wk old; HFK Bio-Technology, Beijing, China) were randomly divided into the following four groups (*n* = 10): the control group; the OHSS group; the OHSS + 16 IU VD3 group; and the OHSS + 24 IU VD3 group. To establish an OHSS mouse model, ovarian induction and VD3 administration were carried out as previously described by Jiang *et al.* and Burjiah *et al.* (2022) respectively. Mice in the control group were first subcutaneously injected with 10 IU pregnant mare serum gonadotrophin (PMSG). Following 48 h, mice were then injected subcutaneously with 10 IU hCG (both from Solarbio, Beijing, China). Both hormones were dissolved in normal saline. Mice in the OHSS group were daily injected with 20 IU PMSG for three consecutive days, followed by injection of 30 IU hCG. Finally, mice in the OHSS + VD3 group received daily 16 or 24 IU VD3 (Solarbio) orally via a gastric probe for 20 d after hCG injection. All mice were euthanized by cervical dislocation following anesthesia with 1.5% pentobarbital sodium. Blood was collected by cardiac puncture. In addition, the ovaries were removed, weighted, and photographed.

### Permeability assay

Permeability assay was performed as previously described (Huang *et al.* 2022). Briefly, mice were injected with 0.1 mL Evans blue (5 mM; MilliporeSigma, Merck, Darmstadt, Germany) through the caudal vein after anesthesia. Following Evans blue injection for 30 min, 2 mL sterile saline was injected into the abdominal cavity and the entire abdomen was gently massaged for 1 min. Peritoneal fluid was extracted and centrifuged at 160 × g for 5 min. The supernatant was transferred into a 96‐well plate and the optical density (OD) was measured at wavelengths of 405 and 620 nm.

### Cell isolation and treatment

The ovaries were first placed in pre-cooled PBS, and following excess tissue removal, they were then incubated in pre-cooled DMEM-F12 (Gibco; Thermo Fisher Scientific, Shanghai, China). To release ovarian granulosa cells into the culture medium, the follicles were punctured with a 1-mL syringe needle. Granulosa cells were then transferred into T25 culture flasks and cultured in DMEM-F12 supplemented with 10% fetal bovine serum (Gibco; Thermo Fisher Scientific) and 1% penicillin–streptomycin (Biosharp, Hefei, China) at 37 °C in an incubator with 5% CO_2_. When confluence reached ~ 90%, the cells were collected after trypsinization and seeded at a passage ratio of 1:3 into a 6-well plate at a density of 10^6^ cells/well. Cells at passages 2 ~ 4 were used in this study. For the subsequent mechanistic studies, granulosa cells were transfected with a PTX3 overexpression plasmid. For cell transfection, pcDNA3.1 plasmids purchased from GenePharma Co., Ltd. (Shanghai, China) were mixed with Lipofectamine 2000 (Invitrogen; Thermo Fisher Scientific, Shanghai, China) and were then incubated for an additional 24 h prior harvesting. In the indicated groups, granulosa cells were treated with 10 IU hCG or VD3 (0, 50, 100 or 200 nM) for 24 h.

### Hematoxylin–eosin (H&E) staining

Ovarian tissues were fixed in 4% paraformaldehyde overnight, dehydrated with gradient alcohol, embedded in paraffin, and cut into 4-μm-thick sections. Subsequently, the deparaffinized tissue sections were stained with H&E solution (Sangon, Shanghai, China) for 5 min and 2 min, respectively, at room temperature. The sections were then dehydrated with gradient alcohol and became transparent using xylene. The tissue specimens were observed under a microscope (Olympus Corporation, Tokyo, Japan).

### Assessment of inflammatory factors

Ovarian tissues were rinsed with pre-cooled PBS (0.01 M; pH = 7.4), and after weighing, they were cut into small sections. Subsequently, the tissues were fully ground on ice and the resulted homogenate was centrifuged at 5000 × g for 10 min to obtain the supernatant. The levels of TNF-α, IL-1β, and IL-6 were measured in the cell supernatant using the corresponding commercially available ELISA kits (Elabscience, Wuhan, China). The OD values at a wavelength of 450 nm were recorded and the cytokine levels were calculated according to the standard curve.

### Serum hormone and growth factor detection

The serum and tissue hormone estradiol (E2) and progesterone (Prog) levels and those of VEGFA were measured with the corresponding commercially available ELISA kits (Elabscience). The OD was recorded at a wavelength of 450 nm and the levels of E2, Prog, and VEGFA were calculated based on the standard curve.

### Western blot analysis

Ovarian tissues and cells were lysed in RIPA buffer and the protein extracts were then separated by SDS-PAGE. The separated proteins were then transferred onto PVDF membranes, followed by blocking with 5% skim milk. The membranes were first incubated with primary antibodies against VEGFA (sc-7269; 1:500), PTX3 (sc-373951; 1:200), and GAPDH (sc-365062; 1:200; all from Santa Cruz Biotechnology, Shanghai, China) at 4 °C overnight and then with the corresponding HRP-conjugated goat anti-mouse IgG secondary antibody (31,430; 1:20,000; Thermo Fisher Scientific, Shanghai, China) for 1 h at room temperature. The blots were visualized using an ECL reagent (Affinity, Changzhou, China) and were semi-quantified using ImageJ software.

### Immunohistochemistry (IHC) and immunofluorescence (IF) assays

For antigen retrieval, paraffin-embedded ovarian sections were deparaffinized and microwaved in 10 mM sodium citrate (pH 6.0). Endogenous peroxidase activity was blocked with 3% hydrogen peroxide. Subsequently, the cells were fixed with 4% paraformaldehyde and permeabilized with 0.5% Triton X-100 for 20 min. Sections and cells were blocked with 5% goat serum for 30 min, followed by incubation with primary antibodies against CD31 (ab281583; 1:4000; Abcam, Shanghai, China) or VEGFA (sc-7269; 1:300; Santa Cruz Biotechnology) at 4 °C overnight. The following day, the cells and tissue sections were incubated with biotinylated goat anti-rabbit (31,820; 1:2000) or FITC-conjugated anti-mouse (F-2761; 1:2000) secondary antibodies (Thermo Fisher Scientific). The specimens were counterstained with hematoxylin and the positive immunostaining was assessed under a microscope (Olympus Corporation). Cells were stained with DAPI and observed under a fluorescence microscope (Olympus Corporation).

### Molecular docking

The molecular structure of PTX3 (PDB ID:7zl1) was downloaded from RCSB PDB (www.rcsb.org). Water molecules and excess ligands around PTX3 protein were deleted, the structure of VD3 was hydrogenated, and systematic three-dimensional structural optimization was then performed. Docking was run in AutoDock software (version 4.2; Scripps Research Institute, San Diego, CA) and the composite structure was displayed.

### Statistical analysis

All data are expressed as the mean ± SD. The differences between multiple groups were compared with one-way ANOVA followed by Tukey’s post hoc test. All data were analyzed using SPSS software. *p* < 0.05 was considered to indicate a statistically significant difference.

## Results

### Effects of VD3 on OHSS pathology and inflammatory responses in mice

**Figure 1.**
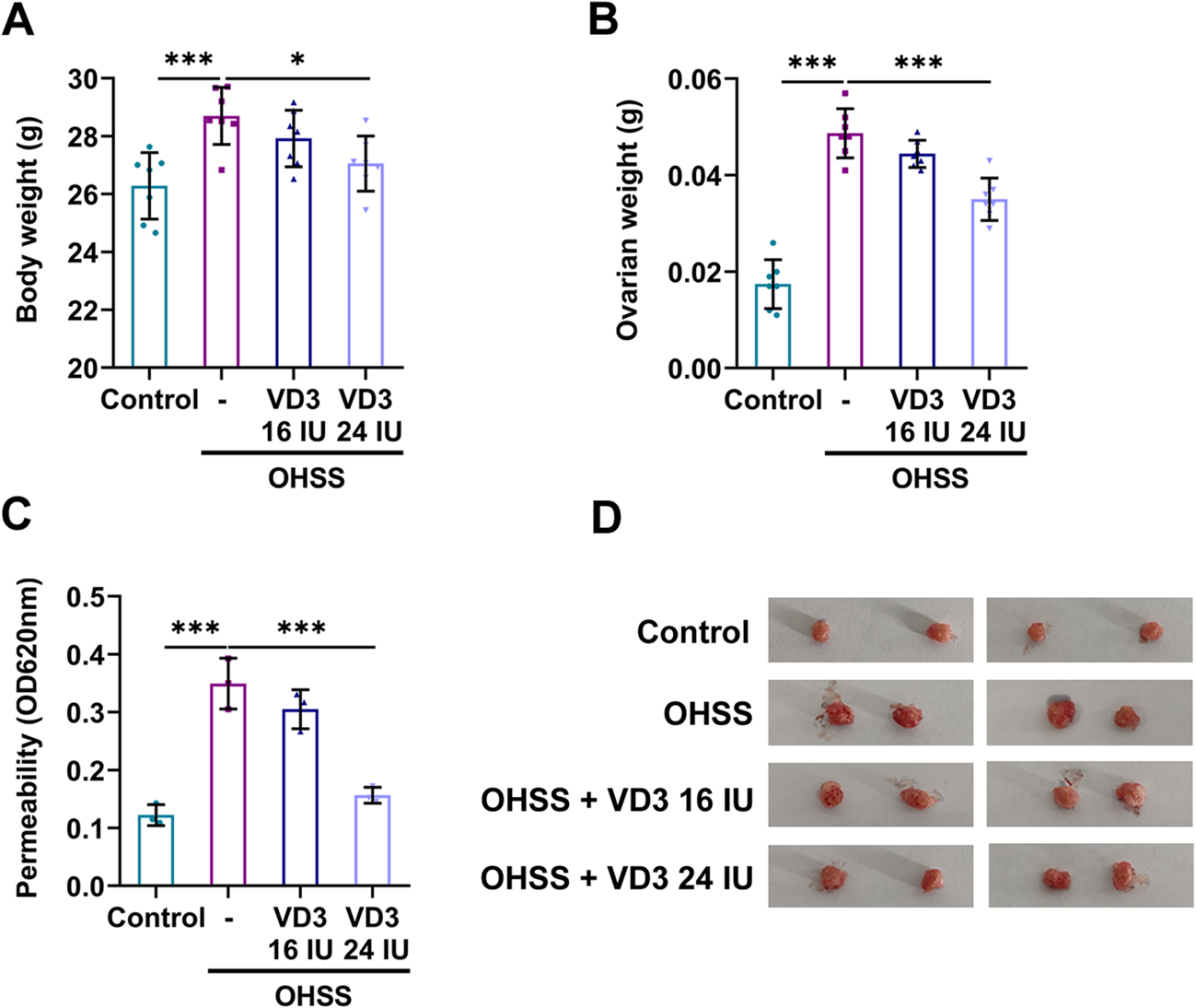
Roles of VD3 in ovarian weight and permeability in mice with ovarian hyperstimulation syndrome. The effects of VD3 on mouse (*A*) body and (*B*) ovarian weights. (*C*) Ovarian permeability was assessed by Evans blue staining. (*D*) Images of murine ovaries were captured. ^*^p < 0.05, ^***^p < 0.001. VD3, vitamin D_3_.

**Figure 2.**
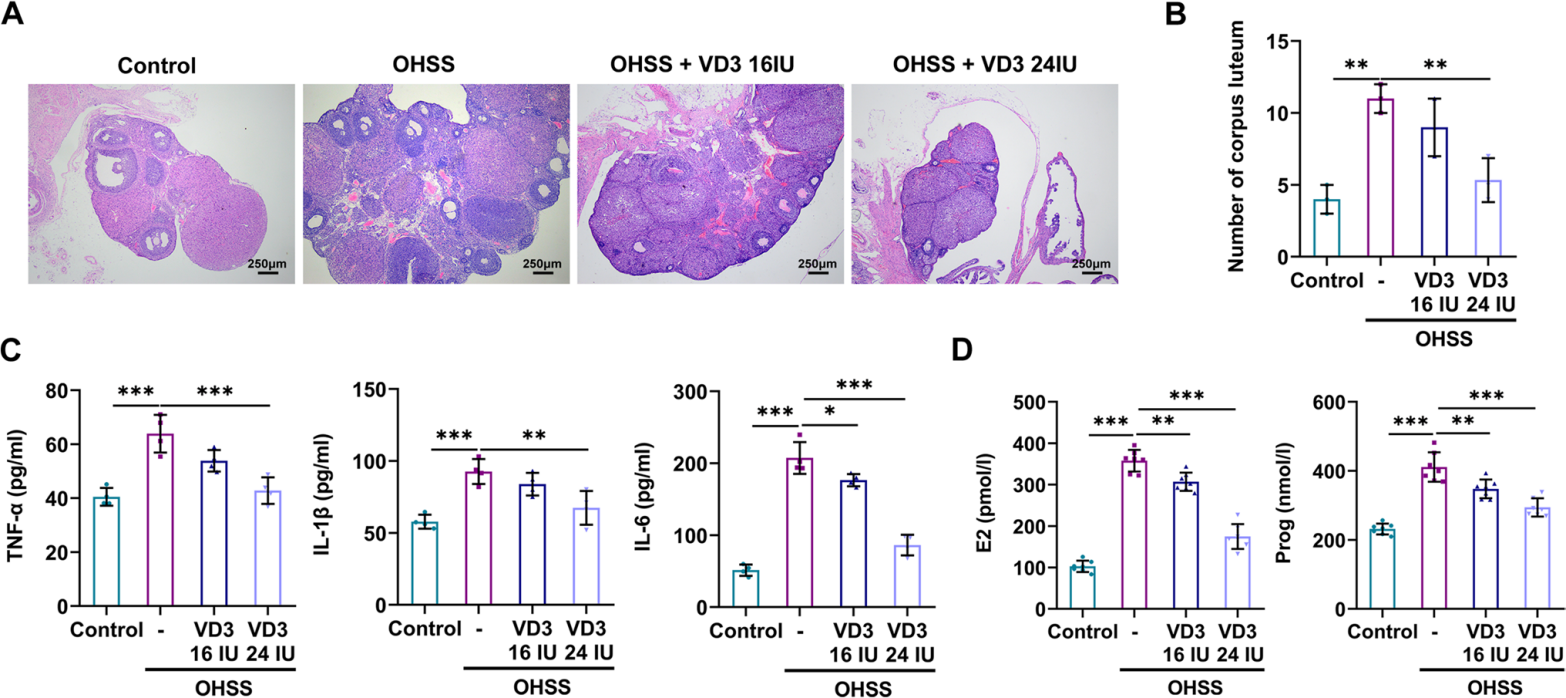
Roles of VD3 in pathological and inflammatory responses in mice with ovarian hyperstimulation syndrome. (*A*) The effects of VD3 administration on the pathological changes of ovarian tissues were evaluated using H&E staining. (*B*) The number of corpus luteum was also counted by H&E staining. (*C*) The effects of VD3 administration on the secretion levels of inflammatory factors in ovarian tissues are shown. (*D*) The effects of VD3 administration on the serum levels of estradiol and progesterone were also determined. ^*^p < 0.05, ^**^p < 0.01, ^***^p < 0.001. VD3, vitamin D_3_; H&E, hematoxylin–eosin.

Significant difference in terms of body weight between mice in the OHSS and OHSS + VD3 groups was observed on the last day. The body weights of mice in the OHSS group were increased compared with those in the control group. However, body weights were significantly decreased in the 24 IU VD3 group compared with those in the OHSS group (Fig. 1*A*). The above changes were more pronounced in ovarian weights. Therefore, the ovarian weights were elevated by approximately two folds in the OHSS group compared with the control group. However, administration of 24 IU VD3 displayed a significant effect on reducing ovarian weight (Fig. 1*B*). Furthermore, the ovarian permeability assays showed that permeability was significantly enhanced in the OHSS group compared with the control group. No significant difference was observed in terms of permeability in the OHSS group compared with the 16 IU VD3 group. However, mice treatment with 24 IU VD3 notably reduced permeability (Fig. 1*C*). In addition, ovarian size was increased in the OHSS group, which was restored by 24 IU VD3 (Fig. 1*D*). Ovarian enlargement and increased permeability are the primary pathological changes of OHSS. The results indicated that 24 IU VD3 could markedly reduce the above pathological characteristics. Furthermore, H&E staining revealed that compared with the control group, the number of corpus luteum and follicles was increased in the OHSS group, accompanied by bleeding and inflammatory cell infiltration. The same findings were obtained in both the 16 IU VD3 and 24 IU VD3 groups (Fig. 2*A*). Statistical analysis demonstrated that the number of corpus luteum in the OHSS group was significantly higher compared with that in the control group, while the administration of 16 IU VD3 had no significant effect on the number of corpus luteum. However, 24 IU VD3 markedly decreased the number of corpus luteum (Fig. 2*B*). Additionally, the secretion levels of TNF-α, IL-1β, and IL-6 were notably increased in ovarian tissues of mice in the OHSS group compared with the control group. Administration of 16 IU VD3 only significantly reduced IL-6 levels, while 24 IU VD3 markedly diminished the levels of all inflammatory factors (Fig. 2*C*). In addition, the levels of E2 and Prog hormones were elevated in the serum of mice in the OHSS group, which were restored by both 16 and 24 IU VD3 (Fig. 2*D*). Increased follicles and corpus luteum are manifestations of ovarian enlargement, and inflammatory cytokines can dilate capillaries and increase permeability. Therefore, the aforementioned findings indicated that the effects of 24 IU VD3 on reducing pathological changes in OHSS were more potent compared with those of 16 IU VD3.

### Roles of VD3 in angiogenesis and PTX3 in OHSS mice

**Figure 3.**
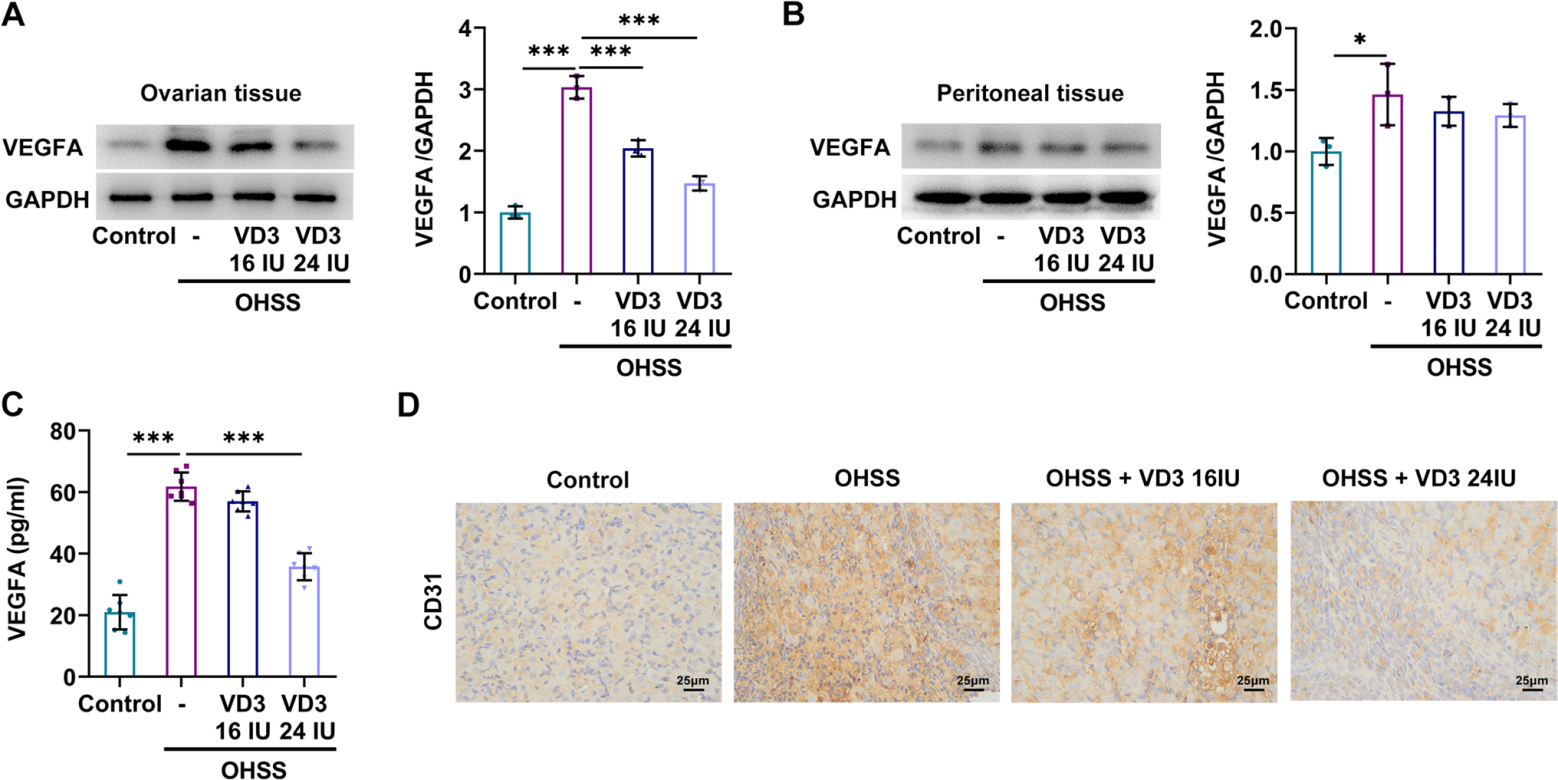
Roles of VD3 in angiogenesis in mice with ovarian hyperstimulation syndrome. The effects of VD3 administration on the levels of vascular endothelial growth factor in (*A*) ovarian and (*B*) peritoneal tissues, and (*C*) serum are presented. (*D*) Immunohistochemical analysis was carried out to measure the levels of CD31 in ovarian tissues. ^*^p < 0.05, ^***^p < 0.001. VD3, vitamin D_3_.

**Figure 4.**
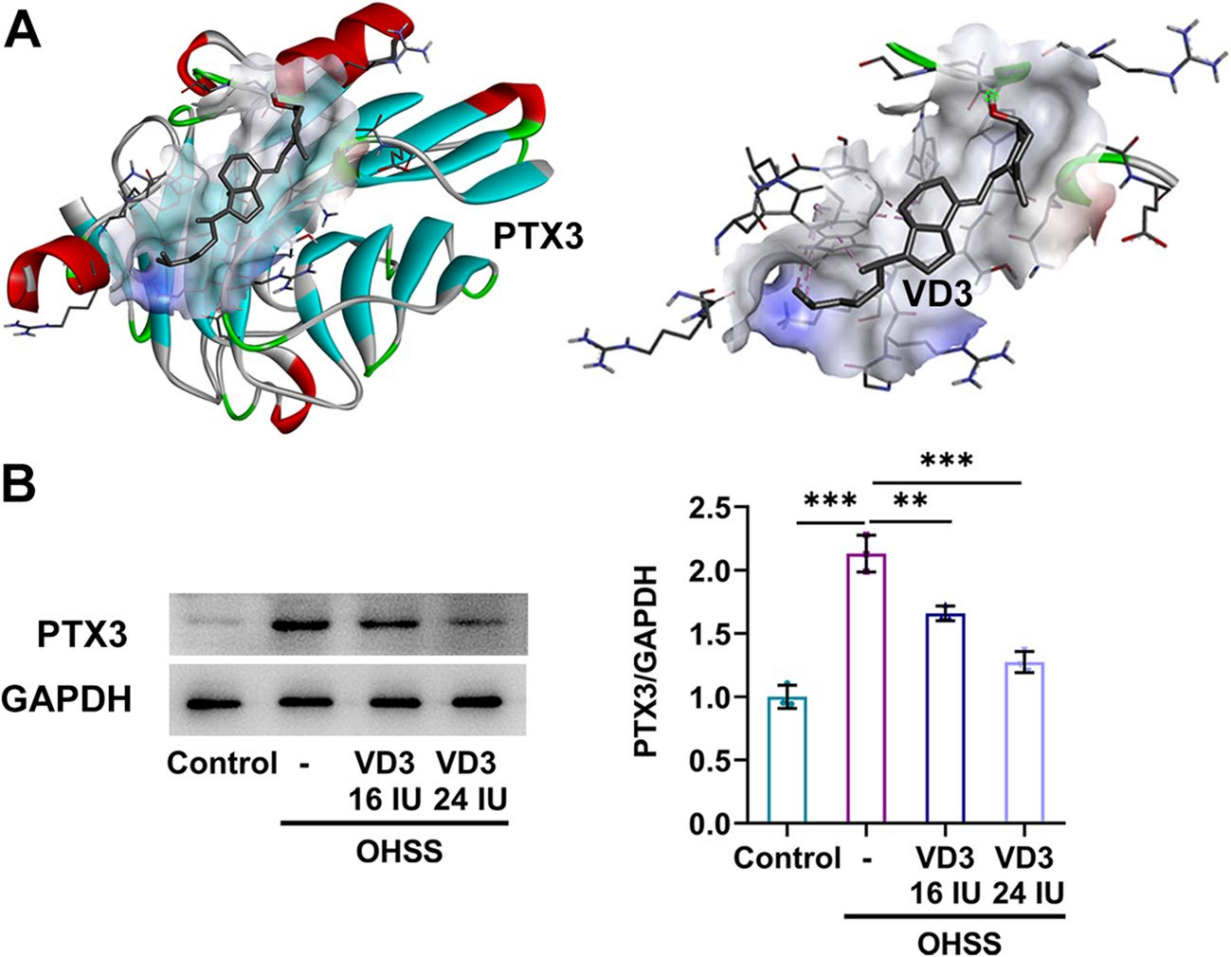
Roles of VD3 in PTX3 expression in mice with ovarian hyperstimulation syndrome. (*A*) Molecular docking predicted that PTX3 could be a potential target of VD3. (*B*) The effects of VD3 administration on the levels of PTX3 in ovarian tissues are shown. ^**^p < 0.01, ^***^p < 0.001. VD3, vitamin D_3_; PTX3, pentraxin 3.

Western blot analysis revealed that VEGF was significantly upregulated in both ovarian and peritoneal tissues in the OHSS group compared with the control group and this elevation was more obvious in ovarian tissues. However, treatment with 16 IU or 24 IU VD3 could effectively reduce VEGF expression in ovarian tissues, but not in peritoneal ones (Fig. 3*A*, *B*). Additionally, the serum levels of VEGFA were also notably enhanced in the OHSS group compared with those in the control group. However, 16 IU VD3 had no effect on serum VEGFA levels, which were markedly reduced following treatment with 24 IU VD3 (Fig. 3*C*). IHC staining assay demonstrated that the CD31 positive rate in the OHSS group was higher compared with that in the control group, which was reversed by 24 IU VD3 treatment (Fig. 3*D*). VEGFA promotes angiogenesis, which is the pathological basis of OHSS, and CD31 has been identified as a marker of high angiogenesis. The above results indicated that 24 IU VD significantly inhibited angiogenesis. Furthermore, molecular docking predicted that VD3 could bind to PTX3 protein through intermolecular forces, thus suggesting that VD3 could possibility act on PTX3 (Fig. 4*A*). Subsequently, western blot analysis showed that PTX3 was notably upregulated in the ovarian tissues in the OHSS group compared with the control group, which was then significantly downregulated by 16 IU and 24 IU VD3 treatment (Fig. 4*B*).

### Roles of PTX3 in VD3-treated granulosa cells

**Figure 5.**
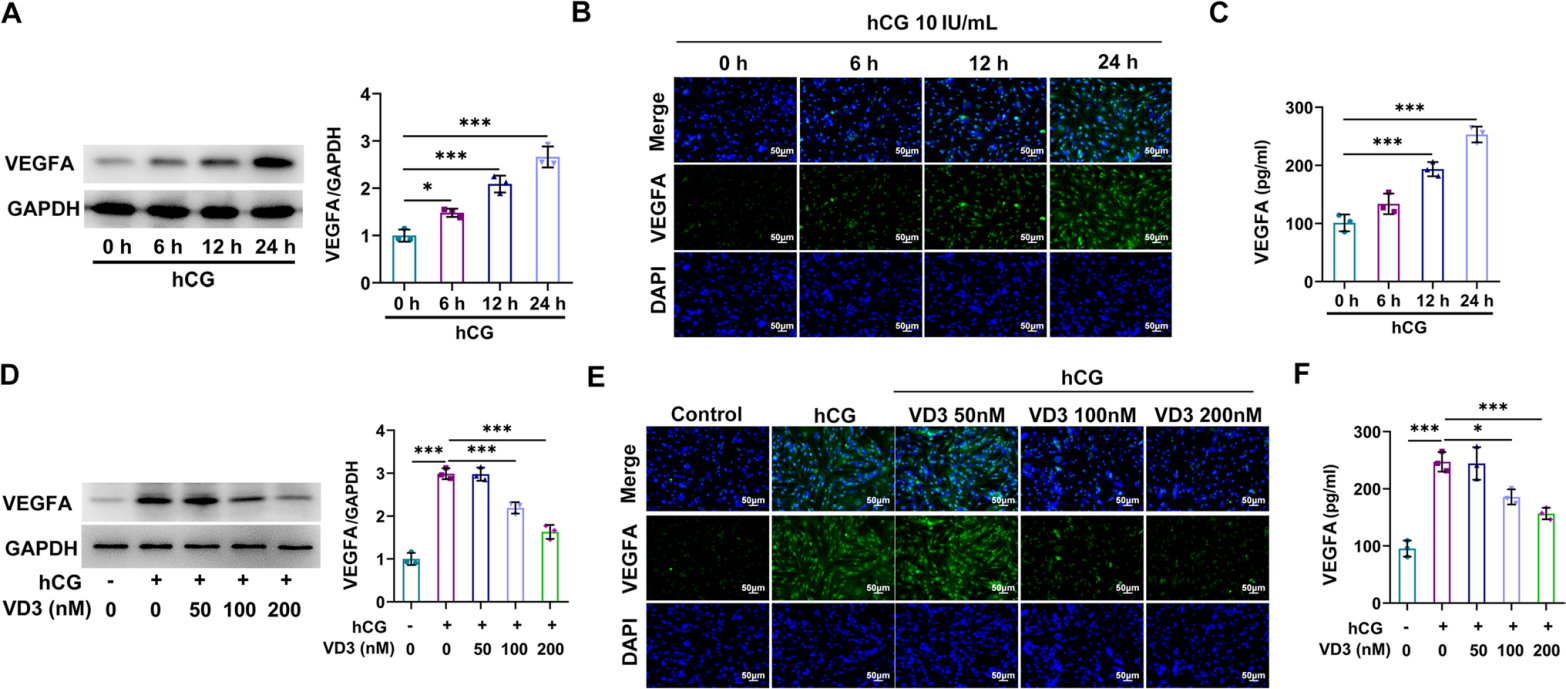
Effects of hCG and VD3 on granulosa cells. (*A*) Granulosa cells were stimulated with hCG for 0, 6, 12, and 24 h, and the protein expression levels of VEGFA in granulosa cells were determined using western blot and (*B*) IF analysis. (*C*) VEGFA levels in cell supernatants were measured by ELISA. (*D*) Granulosa cells were additionally treated with VD3 at a concentration of 50, 100, and 200 nM, and the levels of VEGFA in cells were detected using western blot and (*E*) immunofluorescence analyses. (*F*) The levels of VEGFA in cell supernatant were measured by ELISA. ^*^p < 0.05, ^***^p < 0.001. hCG, human chorionic gonadotropin; VD3, vitamin D_3_; VEGF, vascular endothelial growth factor.

**Figure 6.**
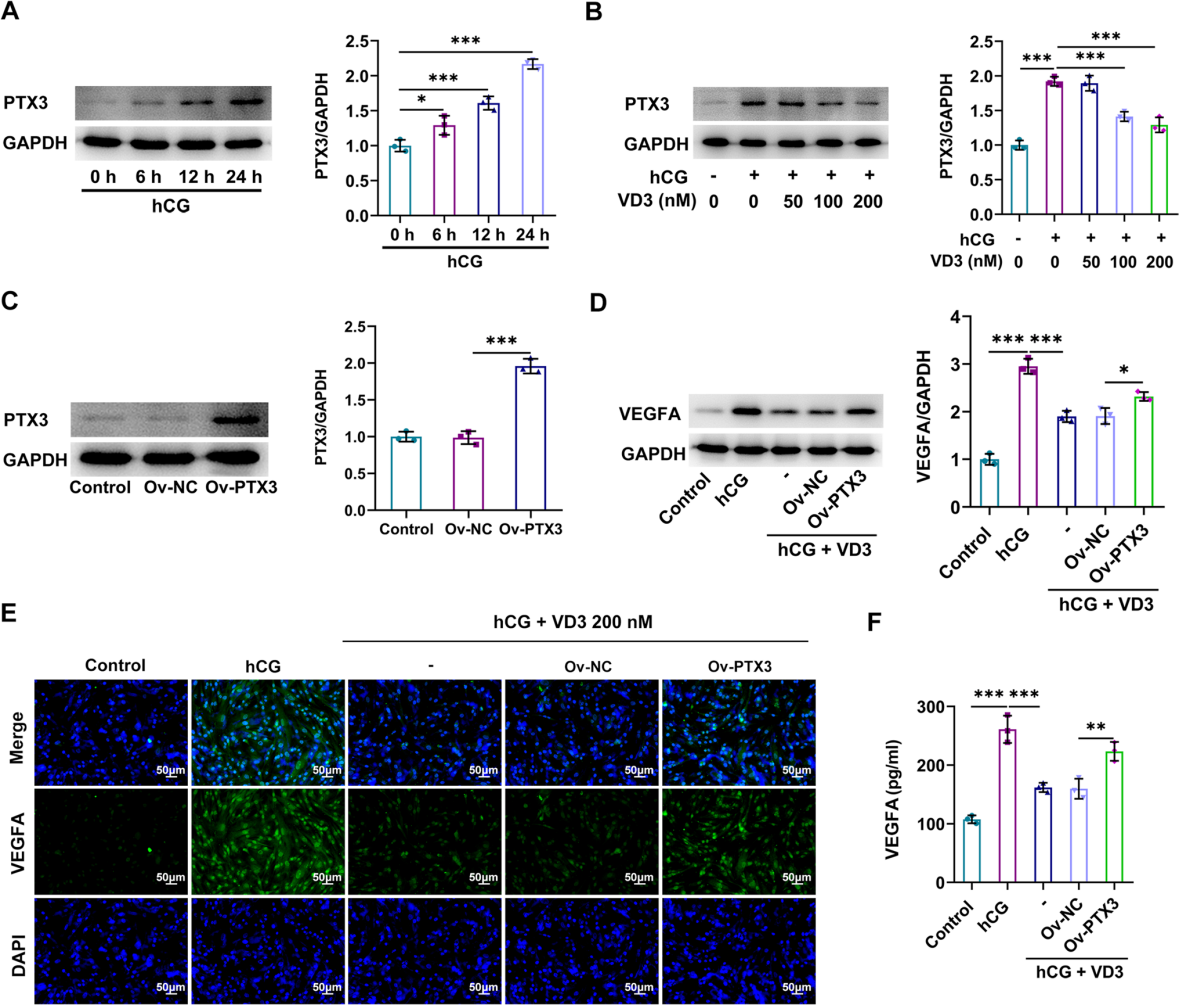
Effect of PTX3 on VD3-treated granulosa cells. (*A*) Granulosa cells were induced by human chorionic gonadotropin for 0, 6, 12, and 24 h and the protein expression levels of PTX3 in granulosa cells were determined by western blot analysis. (*B*) Granulosa cells were treated with 50, 100, and 200 nM VD3 and the protein expression levels of PTX3 were detected by western blot analysis. (*C*) The transfection efficiency in cells transfected with a PTX3 overexpression plasmid was verified by western blot analysis. (*D*) The effects of PTX3 overexpression on the protein levels of VEGFA in granulosa cells were measured using western blot and (*E*) immunofluorescence analyses. (*F*) The levels of VEGFA in cell supernatants were measured by ELISA. ^*^p < 0.05, ^**^p < 0.01, ^***^p < 0.001. PTX3, pentraxin 3; VD3, vitamin D_3_.

Following granulosa cell induction with hCG for 0, 6, 12, and 24 h, western blot analysis, IF assays (Fig. 5*A*, *B*), and ELISA (Fig. 5*C*) revealed that VEGFA was upregulated in a time-dependent manner. Subsequently, following treatment with hCG for 24 h, granulosa cells were co-treated with 50, 100, and 200 nM VD3 and the levels of VEGFA were then determined. Cell treatment with 50 nM VD3 had no effect on VEGFA levels, which were concentration-dependently decreased in the 100 and 200 nM VD3 groups (Fig. 5*D*, *E*). The fluctuations in VEGFA levels in cell supernatants from each group measured by ELISA were consistent with those observed in the western blot and IF assays (Fig. 5*F*). Furthermore, PTX3 was also upregulated in a time-dependent manner following cell treatment with hCG (Fig. 6*A*), which was restored following cell co-treatment with 100 and 200 nM VD3 (Fig. 6*B*). To investigate the role of PTX3, PTX3 was overexpressed in granulosa cells transfected with a PTX3 overexpression plasmid. The transfection efficacy was verified by western blot analysis (Fig. 6*C*). Compared with the negative control group, PTX3 overexpression enhanced VEGFA production and secretion, thus suggesting that it could restore the VD3-mediated VEGFA inhibition (Fig. 6*D*–*F*).

## Discussion

The present study suggested that VD3 could regulate VEGFA through PTX3 in OHSS. More specifically, treatment of OHSS mice with VD3 relieved ovarian pathological changes and inflammatory responses, and normalized hormone levels. In addition, PTX3 was overexpressed in isolated murine ovarian granulosa cells and the results revealed that PTX3 overexpression could affect the VD3-mediated VEGFA inhibition. OHSS represents a major clinical challenge in assisted reproductive technology, with severe cases being life-threatening for patients (Sun *et al.* 2020). Therefore, preventive clinical measures are imperative for the prevention and management of this condition (Timmons *et al. *2019; Castillo *et al. *2020). Although the particular etiology and the mechanism underlying the pathogenesis of OHSS remain elusive, several studies have indicated that VEGF can enhance vascular permeability. Notably, a previous study suggested that enhanced VEGF concentrations in the ascites of patients with OHSS could serve a critical role in the pathophysiology of this syndrome (Farkas *et al.* 2020).

Furthermore, the physiological role of hCG in increasing VEGF expression in granulosa cells during the luteal phase has been well-documented (Fang *et al.* 2019; Wang *et al.* 2021). The pivotal function of VEGFA in promoting angiogenesis and increasing vascular permeability to maintain blood supply to the corpus luteum has highlighted its significance in follicular growth and luteum function (Guzmán *et al.* 2023). It has been also reported that the excessive secretion of VEGF by hormone-stimulated follicles can significantly increase VEGF levels beyond the physiological threshold, thus triggering elevated vascular permeability and the subsequent onset of OHSS (Li *et al.* 2019; Luo *et al.* 2021; Mills and Dahan 2022). This finding was also supported by the results of the current study, demonstrating that the serum levels of E2 and Prog were significantly increased in mice with OHSS. The rise in VEGFA was more pronounced in ovarian tissue compared with peritoneal tissue. VD3 significantly decreased VEGFA in ovarian tissue, while VEGFA in peritoneal tissue decreased slightly. We speculate that this is partly due to higher baseline levels of VEGFA in ovarian tissue, along with higher enrichment of receptors or protein signals affected by VD3 in the ovary. To the best of our knowledge, there is no published data comparing the gene expression differences between the two tissues in the context of OHSS disease; hence, the detailed mechanism deserves further exploration.

The present study further elucidated the role of VD3 in regulating PTX3 expression. VD3 is thought to work mainly through the VD receptor; however, this study discovered that PTX3 is also a potential target of VD3. Based on RNA-seq sequencing consensus datasets (www.proteinatlas.org) derived from HPA and GTEx databases (Uhlén *et al.* 2015), we found that PTX3 was significantly enriched in the ovary compared with VD receptor, approximately seven-fold. This finding supports PTX3 as a vital target of VD3 in regulating OHSS herein. A previous study in transgenic mice highlighted the pivotal effect of PTX3 on innate resistance to pathogens and regulation of inflammation, thus supporting its potential therapeutic role in the management of immunocompromised patients and various inflammatory conditions (Doni *et al.* 2016). Peripheral blood transcriptome results in patients with OHSS and subsequent Gene Ontology and Kyoto Encyclopedia of Genes and Genomes pathway enrichment analyses revealed that differentially expressed genes in high- and low-risk patients were mainly enriched in several pathways, such as the “immune system” (Yan *et al.* 2023). The above results indicated that PTX3 could be considered a therapeutic target for high-risk patients with OHSS and highlighted the potential of VD3 as a therapeutic strategy for the aforementioned patients. Furthermore, the strategic positioning of PTX3 at the blood-retinal barrier interface in the context of ocular inflammatory diseases has underscored its multifaceted role in maintaining ocular homeostasis, thus providing promising prospects for targeted therapeutic interventions (Stravalaci *et al.* 2021).

In summary, the results of the current study provided novel insights into the beneficial effects of VD3 administration on ameliorating the symptoms of OHSS. In addition, the PTX3-mediated inhibition of angiogenic factor release from granulosa cells further supported the potential of VD3 as a novel therapeutic approach for the clinical prevention and management of OHSS. However, the mechanism behind the differential regulation of VEGFA by VD3 in different tissues remains unresolved, and the potential competitive relationship between PTX3 and VD receptor is also unknown, which are the limitations of this study. Further studies should be conducted to fully elucidate the precise molecular mechanisms underlying the interaction between VD3, PTX3, and VEGFA in OHSS, thus paving the way for the development of tailored therapeutic strategies.

## Data Availability

All data generated or analyzed during this study are included in this article.
